# New actions and land uses in the historical heritage: the case study of one of the oldest underground water tanks in Madrid (Spain)

**DOI:** 10.1016/j.heliyon.2022.e12470

**Published:** 2022-12-21

**Authors:** María Jesús Rosado-García, Susana López-Querol, Daniel Crespo Delgado

**Affiliations:** a Departamento de ingeniería y gestión forestal y ambiental, Universidad Politécnica de Madrid, Spain; b Department of Civil, Environmental and Geomatic Engineering, University College London, UK; c Fundación Juanelo Turriano–Departamento de Historia del Arte, Universidad Complutense de Madrid, Spain

**Keywords:** Heritage, Hydraulic industrial architecture, Historical masonry structures, Rehabilitation, Water tank

## Abstract

The preservation of architectural heritage in Europe is generally associated with the recovery of monumental buildings such as churches and castles, leaving behind a high amount of other historical constructions of civil and industrial architecture that exist in an old country like Spain. High efforts have been devoted by professionals from very different fields aiming to recover and enhance engineering works such as bridges, but the particular case of hydraulic works deserves special attention because it has remained hidden for centuries and sometimes continues to be ignored by society and many institutions. This type of architectural heritage, in the form of underground deposits that represents the case under study in this research, occupies a large area in cities such as Madrid and must be preserved to relieve the pressures of adapting them to accommodate other uses of the land that they occupy. In this paper, the complexity of material characterization and calculation of the Mayor water tank in Madrid (Spain), as an example of this type of construction, is presented. This reservoir currently supplies drinking water to a large part of the city and can accommodate a park on its surface without altering an original 150-year-old structure. As the main conclusion of this research, and in the absence of detailed studies of pathologies, tests, characterization of materials and the terrain, it is recommended to avoid loads greater than 30 kN/m^2^ in the existing structure, hence the current stresses are not substantially exceeded. This is a recommendation to preserve the historical heritage of this structure against economic and political perspectives that push for a substantial change in its use. This research is an example that can easily be extrapolated to other similar historical infrastructures aiming to guarantee their prevalence in the future.

## 1 Introduction and literature review

Nowadays there is a worldwide growing interest in the recovery of the civil and industrial heritage of old cities, although the unstoppable real estate pressure is also systematically demolishing industrial complexes, or sometimes reusing them without any reasonable or logical criteria. Examples of the disappearance of large industrial monuments are the old Euston Station in London, in 1962, the Halles Centrales in Paris, in 1971 and the great Salamanca Water Reservoir in Spain, demolished in 2002. However, rehabilitation or preservation is currently understood as a form of sustainable and culturally diversified development, thanks to the movement to revalue industrial heritage (Hudson, 1964). The water industry is dealt as World Heritage (Douet, 2018), in particular water tanks that have survived from the past to the present are considered within the scope of industrial heritage and should be highlighted the importance of preserving this heritage. The consolidation of Industrial Archaeology had its beginnings in Great Britain (1962) where valuation criteria were established including four categories that have allowed the identification of the qualities of these constructions for the acquisition of new uses (VVAA, 2008). Industrial heritage is reinterpreted to adapt to the needs of today's society. In addition to these criteria set out by English Heritage on the value of the heritage of Historic Environments and Industrial Sites, in the case of canals, for example, the specific criteria put forward by TICCHI (The International Committee for the Conservation of the Industrial Heritage) (Icomos, 2022).

**Figure 1:**
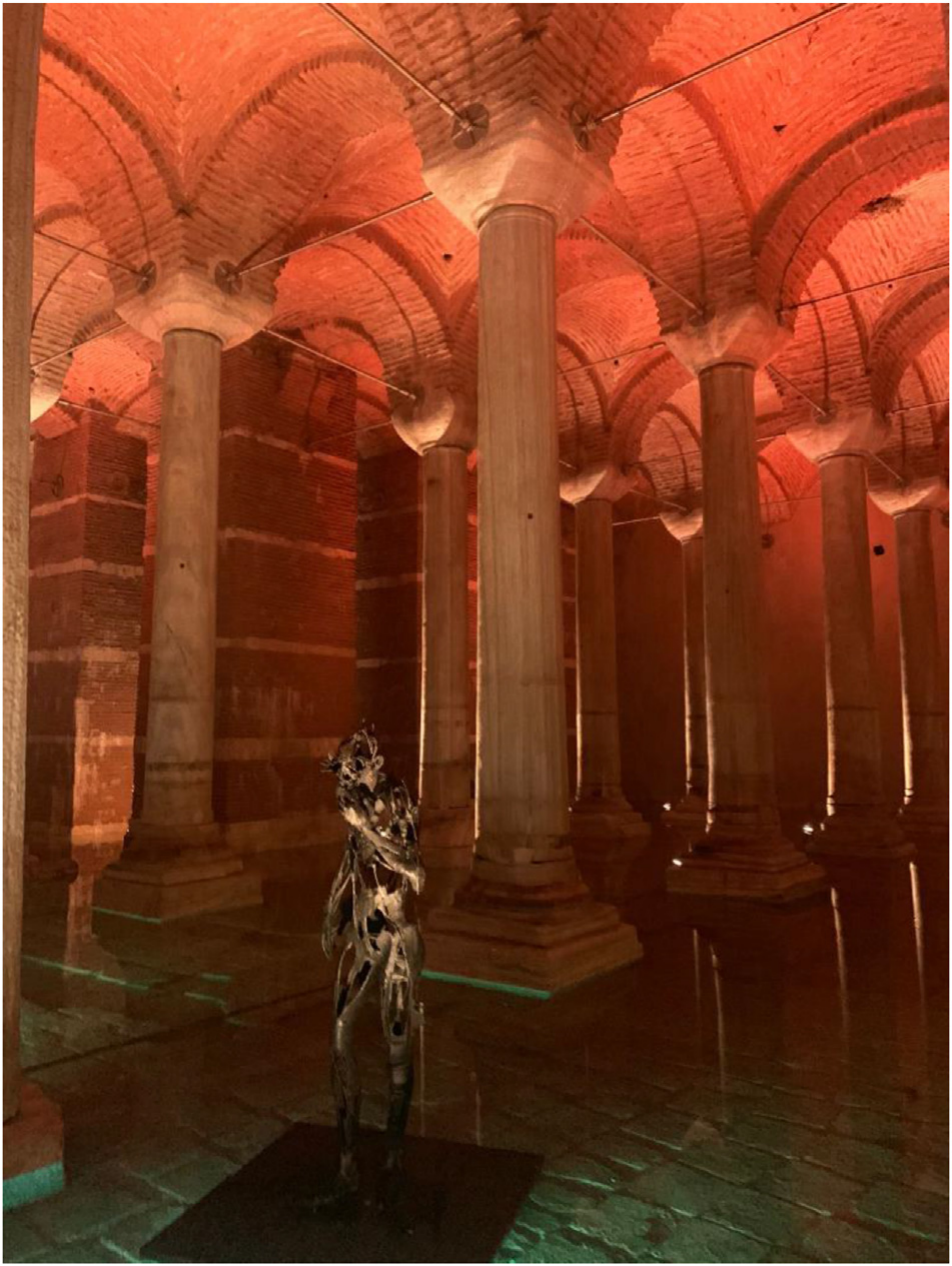
Yerebatan Palace after the restoration works, the authors took the view in August 2022.

There are many benefits to maintaining the existing infrastructure and extracting the maximum value from its assets, which leads to prioritising the rehabilitation of works that could be initially thought of as being demolished, such as the case of the St. Petersburg water tank, which was deteriorated and leaking after of 90 years of use (Bruder and McGrath, 2015). Reported examples of the recovery of this type of heritage are limited in the literature, being mostly individual case studies. It is worth mentioning that these constructions are remarkable examples of the beauty of the connections between the science of water engineering and their architecture (Yilmaz Emre, 2014), such as the Yerebatan reservoir palace in Istanbul (Figure 1) with continuous conservation studies (Aydıngün et al., 2020).

**Figure 2:**
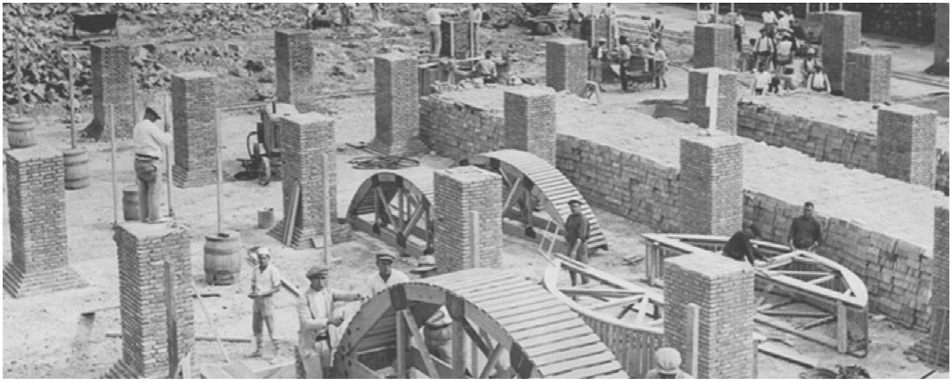
Construction of the Valencia water tank (1849–50) Source: Valencia History Museum, https://mhv.valencia.es (MHV, 2022).

Regarding interventions in buried reservoirs in Spain, it is worth highlighting the Rei Martí reservoir in Barcelona designed by Gaudí (Chalamanc et al., 2017) or the first Valencia water reservoir in Spain (Figure 2), one of the best examples of Valencian industrial architecture from the mid-nineteenth century, now converted into the city's History Museum (Aguilar-Civera, 2004).

On the other hand, the fact that masonry is an ancient material does not mean that its behaviour to withstand different stresses in structures built with it is fully understood yet. Likewise, there are few works (Martinez, 2003) on the study of methodologies that allow homogenising the safety of historic masonry structures, due in large part to the disparity of their constructions, structural elements and constructive particularities of civil or religious buildings, public works such as masonry bridges, fortifications and walls, towers, and other constructions such as lighthouses, port works, dams, canals, reservoirs, etc. Moreover, their pathologies are diverse, although in most cases it is possible to distinguish between those caused by the degradation of materials or the presence of water, or issues of structural or geotechnical origin. As the most common pathologies, we can expect foundation problems that lead to the cracking of some structural elements due to differential settlements.

Spanish hydraulic engineering, and in particular the one carried out in its capital, Madrid, marked a decisive milestone in the 19th century (Rafo, 1849) with the construction of both underground and elevated water reservoirs to respond to the rapid growth of the population and the expansion of the city, developing a modern urban network of quality water supply (Arroyo-Ilera, 2004). It should be noted that the very first origin of the name “Madrid” derives from the Arabic “*Mayrit*” or the place where the Mayra abounds, the water pipes (Asín, 1959).

**Figure 3:**
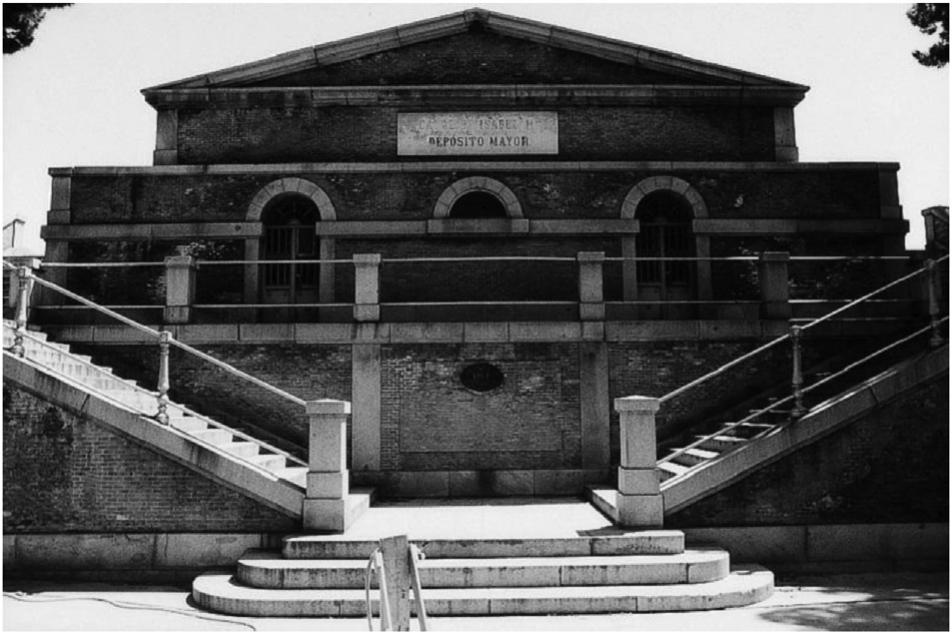
Access to the second underground water tank of the Canal de Isabel II, or Major Tank. Source: https://artedemadrid.wordpress.com/(ArteenMadrid, 2018).

The first water tanks in Madrid were built under the existing urban framework, hidden from the population. The precise location of most of these tanks is still unknown by most, despite their historical and heritage value, as revealed by a exhibition (Aguiló Alonso et al., 2001). There are about 300 water tanks distributed throughout the city, but it is worth highlighting the singularity of the first three that were built, and in particular, the so-called second or Major Water Tank (Figure 3), which is the object of this research. Its construction started in 1863, next to the first tank, to increase its capacity by about 200,000 cubic meters. It is still in use nowadays, being the oldest one in Madrid, as the first went out of service, there is also a debate to propose new uses for it (Saiz, 2021), and it supplies drinking water to a large area of Madrid through the public company Canal de Isabel II (Martínez Vázquez de Parga, 2001).

**Figure 4:**
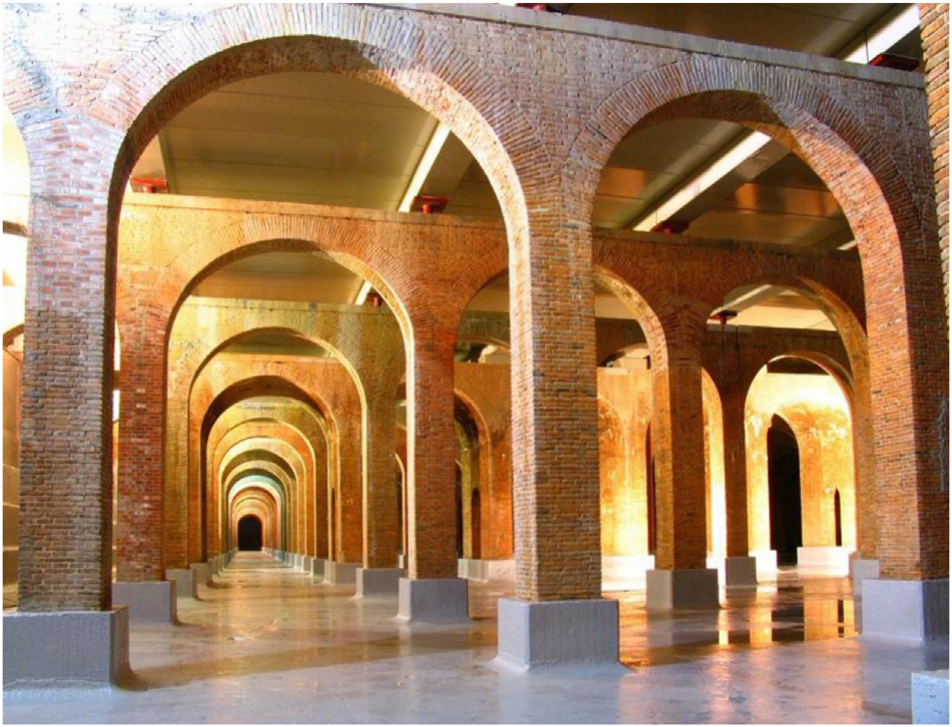
View of the new roof over the original brick arcade structure of the third underground tank. Source: https://www.aguasresiduales.info/(Aguasresiduales, 2022).

Towards the end of the 19th century, the brick arcades of the third tank were designed, aiming at increasing its capacity to supply a population of nearly 600,000 people. This new tank was a cistern with twice the capacity of the second tank, and it was near to the previous ones to connect them and equalize their height. The collapse of the roof of during its construction in 1905 deserves to be mentioned. Despite the extraordinary controversy it caused at the time when concrete was new material in full expansion, the case fell practically into obscurity (Diaz-Pavon, 2016). It should be noted how in this case the adaptation to a new use at the beginning of the 2000s gave rise to a restoration (Figure 4) that altered the integrity of the original construction. Its surface is now employed as a public park and sports facilities, with modern action prevailing over the preservation of the work (Candela, 2007).

There is a current debate about the reconsideration of the superficial use that these constructions occupy in the centre of a city like Madrid, due to social, political and economic constraints. In the case of the second tank, and due to other interests unrelated to the preservation of the work, public media echoed the action before the technical analysis of its viability (Telemadrid, 2016), which in most cases does not give a green light to possible changes of use of the infrastructure, as it will be highlighted later in this paper.

The third deposit, with the demolition of part of its structure to accommodate a golf course on top of it, is an example of how sometimes other interests prevailed over technical and patrimonial benefits, of which the second deposit is a good example because the results and conclusions from the present study were transferred by the authors to the local Administration in 2016 and served as a reference, aiding the preservation of the integrity of the work, as later described in the following sections. In summary, as an alternative to the initial proposal, which consisted of a new part on top, the design was modified to guarantee and priories the prevalence of this iconic infrastructure. Precisely on part of the wall of this deposit, a vertical garden has been installed, made up of 15 different species of plants with a very efficient irrigation system. The park has also space for walks with sustainable paving, 50 trees, nearly 1,000 bushes and landscaped areas not only with grass but also with species with low water consumption requirements (CYII, 2020).

The general objective is to provide examples of the problem to preserve the heritage due to new actions in the cities. A particular objective is to show how typical constructions of underground water tanks in Madrid have a series of common features that make it difficult to technically recommend changes in their use, since in most cases they entail a change in the loads under which they have been working for centuries and for which they were designed. Only the problems in the carrier system of the historical monument will be examined, other proposal will be made in future studies. On top of that, the characterization of the materials they are made of is not easy, due to the consequent deterioration over the years, the heterogeneity of the masonry elements and fillings, as well as their different pathologies caused by the size of the structures, making all of this evaluation very difficult. Therefore, adopting unique and coherent decisions for adapting these infrastructures to new uses is challenging.

**Figure 5:**
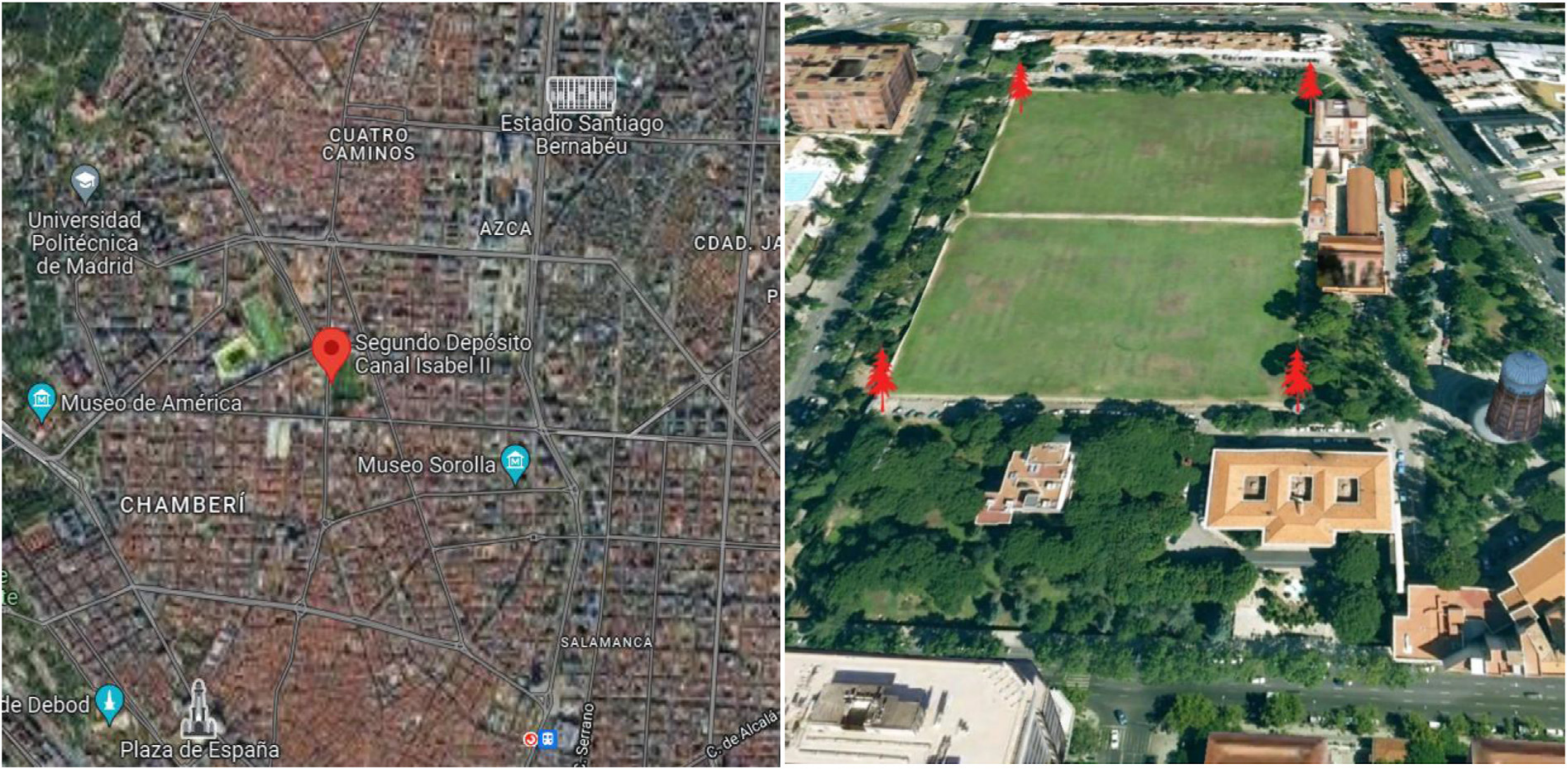
Situation of the second tank (Segundo Depósito Canal Isabel II in Spanish) in the city of Madrid and aerial view after waterproofing in 2012 with the placement of artificial grass on the roof. Source: https://www.google.es/maps/.

The estimation of admissible loads in this study contemplates the definition of different options, i.e. punctual and distributed (CTE, 2006), on the roof of tank number 2 of Canal de Isabel II, located between Bravo Murillo and Santa Engracia streets (Figure 5).

**Figure 6:**
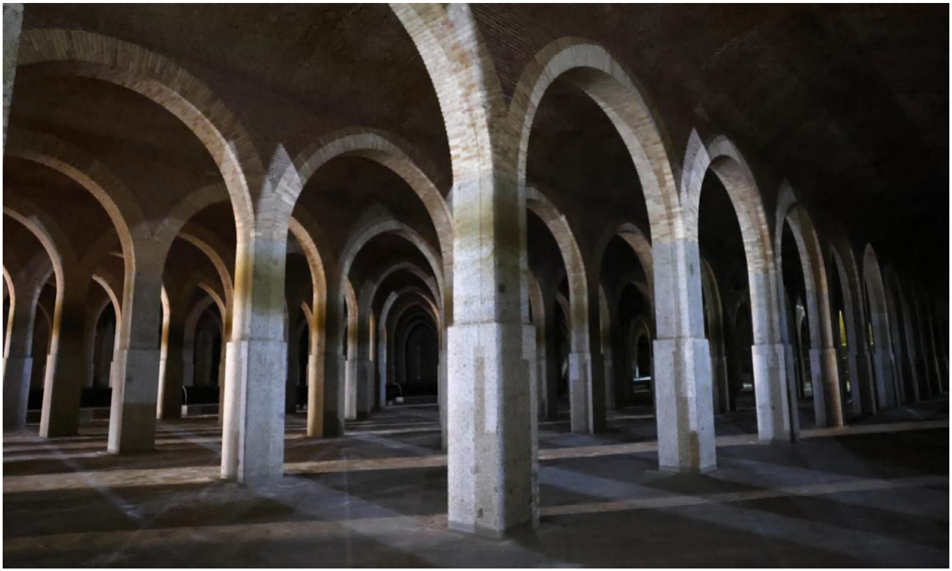
Interior view of the empty second (or Mayor) underground tank during the waterproofing works. Source: https://www.comunidad.madrid/(Madrid, 2021).

The structure of the second tank has a total size of 208 × 138 m^2^ in plan view and consists of 1,040 brick pillars with stone bases and round arches covered by barrel vaults (Figure 6). The objective of this research was to assess the stress state of the vaults and masonry arches that allows analysing of the layout of overloads on the fill, because the construction of a new urban park on its surface, with the consequent increase in loads, was planned. The foregoing entails a stress study of the components of the structure, including the foundation, before and after the last performance of waterproofing the roof in March 2012 (CYII, 2012) as well as after the hypothetical application of loads, both distributed and punctual in the worst possible positions per element that allows obtaining admissible load recommendations for the roof in its current state.

This several years old structure has undergone a number of modifications since its original layout, and therefore it is important to describe, document, and chronologically model each situation, to have a clear track of the stresses in its elements.

The present research entails the development of a 3D finite element model, which reflects as closely as possible the stresses that appear in the different structural elements and materials, namely, earth fill, masonry, granite from the piles, clay and concrete of which is composed in its current configuration. In this way, the stress state is evaluated before (current situation without change of use) and after the actuation (future situation if there is a change of use), considering different load states in the future, allowing us to make efficient decisions to guarantee its preservation under its new use. The visual inspection of the current structural condition, a deep literature review and the characterization of the materials that allow for accurate modelling have been conducted. The main results of this analysis are presented and discussed and the main derived conclusions are listed at the end of this paper.

## 2 General considerations

### 2.1 Mechanical behaviour of the masonry

Works made of masonry have peculiarities and affect their mechanical behaviour. In this case, we are talking about solid bricks, joined by a binder or mortar, which in turn could be of many different typologies. On the other hand, the lock or rigging also presents a multitude of variables. This is the explanation for the great dispersion of the mechanical properties of the fabric under study. It is well known that the fabric is a composite material and relationship between the different properties of materials is important (Mann and Müller, 1982).

On the other hand, the conditions of the fabric are very different from those of the isolated pieces. In addition, as indicated above, it is important to know the current conditions of the masonry, as some parts can be more deteriorated than others due to different histories of stress.

Also, the fabric is a doubly anisotropic material, that is, the fabric has the intrinsic anisotropy of the elements, and also the existence of vertical and horizontal joints (respectively, sores and linens), conditioning its mechanical behaviour in each direction. It is worth noting that, as a common and general characteristic of the fabric, it is practically impossible to develop tensile stresses.

After this basic introduction about the performance of the majority of material that makes up the structure of the deposit, the constituent materials (pieces, mortar and interface) are identified and characterized according to the studied bibliography.

### 2.2 Identification of the elements and their function on the overall structural performance

**Figure 7:**
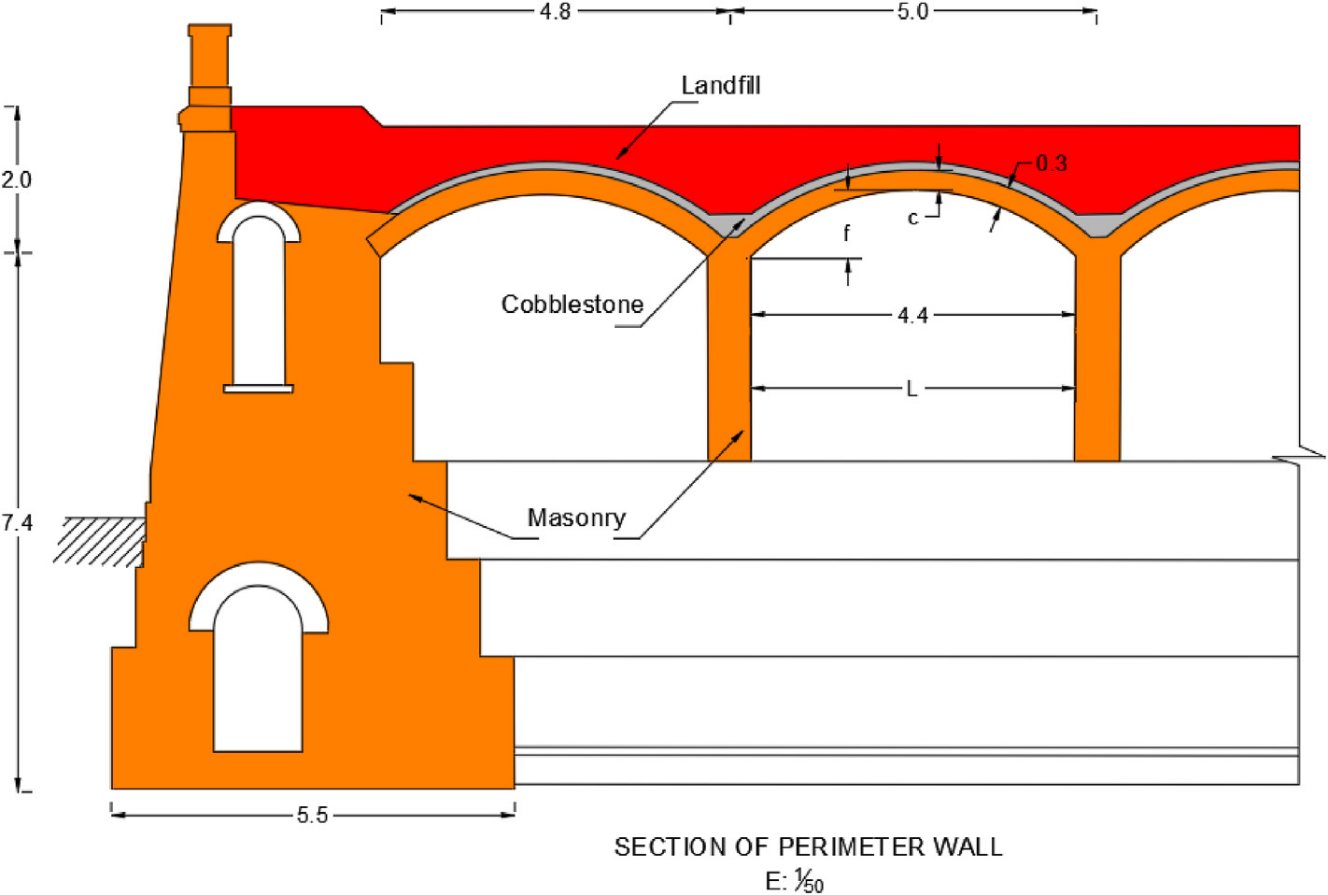
Detail on the perimeter wall and type arch-pier (dimensions are given in m).

In the structure under analysis, several elements can be distinguished. As a preliminary step, it is convenient to identify which of all of them develop a given structural mission. The most relevant structural elements are vaults, fills, arches, piers, foundations and the perimeter walls (Figure 7).

The vault is the main resistant element in the structure under study and has two general properties: it works by its shape and is made with materials that are not capable of withstanding tensile stresses. For a better analysis, we can classify it as a straight vault and three parameters must be defined to obtain a preliminary approximation of its performance: the span (L), which is 4.4 m; the slenderness (c/L) which is quantified through the relationship between the depth of the vault of c = 0.3 m in keystone; and the free light and the rise of the arch (f/L). Given the material used in the tank vault, it is classified as a solid brick masonry vault, bonded with lime mortar, with mixed rigging, with which it is possible to have a preliminary estimation of the mechanical properties of the vault. The term “fill” does not have a clear acceptance in historic structures. In most cases, it refers to everything that is located above the vault, and in this case, it also refers to the material confined between the supporting walls. The water and its seepage are the worst enemies from the durability point of view for these structures due to the washing of joints, vegetation, etc. In this sense, this aspect should not worry us, since after the consolidation and waterproofing of the roof, the presence of water in the fill seems unlikely. This aspect was therefore neglected in the conducted structural analysis.

Lateral confinement of the fills can be understood as the restriction to their horizontal deformation. In the case under study, it seems clear that the fills are transversely limited by the vault itself, the concrete slab on it and the lateral support walls (Figure 7). The physical parameter that defines this state is Poisson's ratio of this material.

The function of the piers in this case is to drive the load transmitted by the vaults and arches to the foundations. In the second tank, these are made up of masonry in their upper part and arches, giving continuity to them once we lose elevation through two different square sections (60 and 70 cm) of granite. From the analysis of its original design, it seems that the progressive increase of the compression of the piers in depth was considered, so the material was changed accordingly to guarantee a higher compressive strength. In the numerical model developed for this research, these stresses are quantified and compared with the strength of the material, to assess whether they would be acceptable or not.

Thus, as in the vaults, the structural behaviour of the piers can be characterized through a few geometric parameters and the type of section described. The relationship between the depth of the pier and the free span of the vaults is the geometric parameter that best characterizes its behaviour, since as a multi-arch structure, there is an interaction between the different arches and vaults, in addition to the piers themselves in the general resistant operation. In this study, the piers are classified as mixed solid masonry and granite. And, as the most common pathologies, we can expect foundation problems that lead to diagonal or vertical cracking in the plane of the pier due to differential settlement.

The foundation is the worst known and weakest resistant element out of the aforementioned elements. There is little documentation about its design and construction compared to, for example, existing design rules for vaults. In addition, it is a hidden and inaccessible element. It is the weakest, because it was built using masonry, and we intuit that it rested over the limited knowledge of the geotechnical properties of the land on which it rests. In this case, the foundation directly transmits the column load to the supposedly competent stratum through a footing, which, according to its dimensions, behaves as rigid.

### 2.3 Uncertainties

The evaluation of the masonry structure under analysis involves a large number of uncertainties. In this specific case, it may be a limiting circumstance, since the distribution of the stresses and strength variables are not fully known, and the tank has undergone a long history and variation of loads and changes in the condition of the materials. Hence, in practice, the uncertainty is high. This fact means that the scope and precision of the analysis are somehow limited, or what is the same, it is necessary capacity for criticism and engineering judgement.

**Figure 8:**
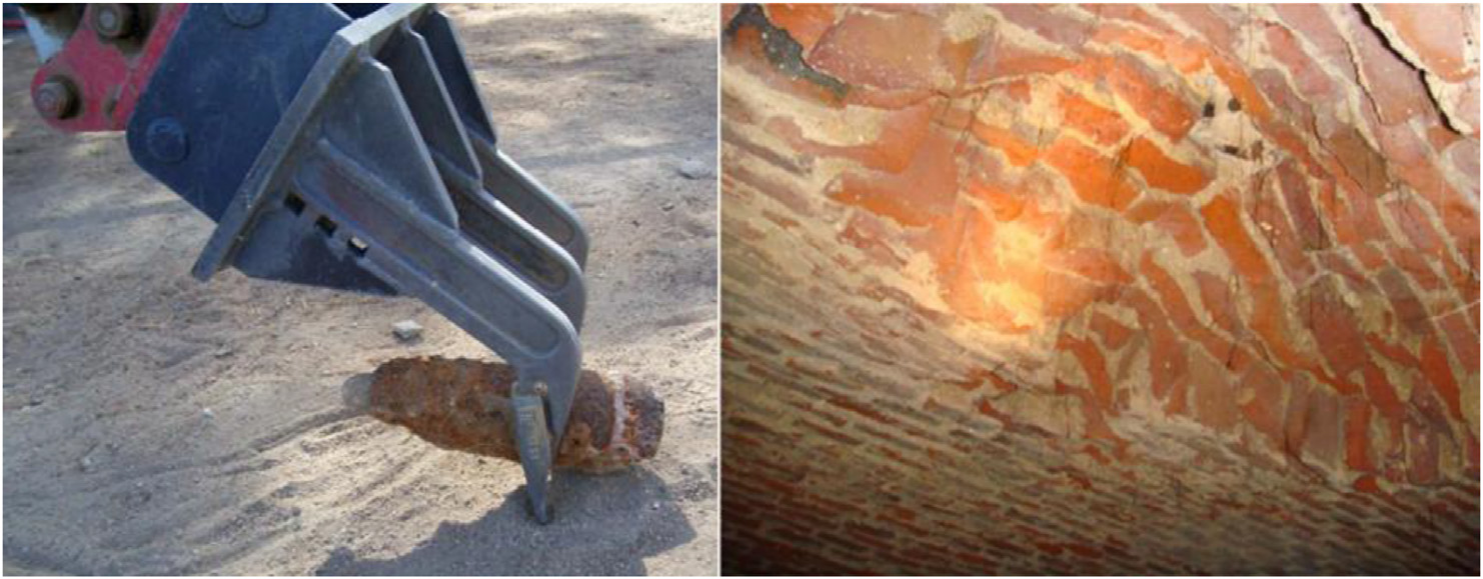
Shell found in the excavation during the operation and the detachment caused in the vault.
• Materials: the greatest uncertainty that we find in masonry structures is the great amalgam of materials used as well as their variability, even within the same structural element. This can cause zones of different resistance and deformability in the same vault or arch.
• Foundation: the knowledge of the type and materials with which the foundation was made is essential, although the original drawings show a 4 m^2^ masonry direct foundation (CYII, 2012). It is worth highlighting that any settlement in the foundation can lead to catastrophic consequences in the structure since the loss of its original shape would be fatal.

Having said that, it is important to be careful not to push the structure beyond its limit, as well as to specify and delve separately into the various uncertainties we encounter. These are:

• Geometry: The geometric data in these structures, almost always reflect quasi-perfect shapes, and do not usually reflect batters, imperfect arches, etc. These facts are extremely important, since, especially in the interpretation of the deformed shape, congenital defects of this type of structure are not taken into account. This can significantly alter the strength configuration of vaults and arches.
• Load: the loads to which this structure has been subjected are not fully known. In the first place, this structure will have been subjected to various loads, due to its long useful life, although it seems that there have been no considerable overloads on the roof, apart from those caused by its regular maintenance. Similarly, it seems that there have been some extreme situations, as can be documented by the appearance of a shell that caused damage inside (Figure 8). There is evidence of its appearance, but not of the magnitude of the caused damage.

The more the previously mentioned uncertainties are reduced, the more accurate the analysis, so it will be possible to evaluate with higher certainty the stress regime.

### 2.4 Characterization of the materials

Unfortunately, the lack of regulations that protect the characterization of the existing masonry very much complicated this study, although there are recommendations and proposals for carrying out tests for new masonry works (P.I.E.T.70, 1971) (EC6, 2011).

The main materials of the tank are brick or masonry and granite. Likewise, the layer of mortar that covers the vault is defined as calicanto, and due to its similarity with lime mortars, their mechanical characteristics have been adopted. Since the construction of the masonry is assumed to have been bound with a specific type of mortar, it is likely that the same binder was used for the upper layer of the vault. To the previous original materials of the structure, those arranged after the waterproofing works of the roof in 2012 are currently added (CYII, 2012). The cover of the deposit was concluded during that action by a 10 cm deep layer of gravel. In addition, gravel was placed in the drains executed every 10 m above the frames. Likewise, the mechanical characteristics of the clay-type concrete layer arranged on the roof must be defined.

The materials are considered elastic, and the comparison with the compressive strength is made by comparison of the elastic stresses obtained. According to the indications of the regulations and previous research (Martinez, 2003), the material properties are:

### 2.5 Characterization of the underneath soils

The geotechnical report carried out in February 2011 was employed to estimate the soil properties (CYII, 2011). That information data is provided only for the North face of the deposit, and the values specified there have been adopted for all the supports of the structure.

## 3 Modeling and methods

In addition to a very detailed bibliographic and documentary research on past actions carried out in this tank, a structural evaluation was conducted through numerical analysis. Methodologically, beginning with the general conditions, we then carried out specific analysis that required our creativity due to the large undetermined loads, properties, and heterogeneity of the materials. Several actions were carried out, such as a visual inspection, with the taking of photographs, which allowed us to draw up a cartography of its state and anomalies. About the previous conditioning factors, this type of intervention in urban and historical heritage shares the same requirements for their execution, such as the need for a study without service cuts. A common conditioning factor in this type of project is the heterogeneity of this type of construction. The composition and properties of the materials, nowadays, have to be estimated. The different aspects of the structural analysis are presented next.

### 3.1 Description of the problem, based on performance-based design

The study of the structural behaviour under serviceability conditions is essential to know the normal working regime of the structure, as well as to allow us to identify the causes of some of the pathologies. In the case of the structure of the tank, any numerical result has a validation element, which is the fact that it is still operational after 150 years. Hence one of the most important criteria to take into account is not exceedance of the stress state to which the structure has worked during its life. For example, problems derived from settlements on the ground due to unusual overloads may cause the structure to collapse, even though the vaults, arches and piers are capable of admitting them. This phenomenon, given the above-listed uncertainties of the problem, is a good starting point.

The analysis has been conducted following the next steps:

• The main structural elements have been geometrically and mechanically identified and characterised.
• The type and frequency of the failures identified so far have been studied, in case they have occurred.
• The found pathologies have been recorded, as well as their influence on the whole structural behaviour.
• The numerical models to analyse the strength of the structure have been developed.
• The most important variables that control the different structural elements have been identified.
• The actual structural layout has been estimated. To do so, the different actions in the original structure have been studied, along with the history of loads that it has been subject to and the possible current mechanical properties of the materials.
• The real stress state, in the past and nowadays, has been identified, in order to plan a possible actuation that guarantees the safety of the structure.

### 3.2 Safety framework

The current safety framework for the calculation of structures is based on the theory of limit states, which is grounded on the comparison between factored stresses (Sd) with the response of the reduced capacity of the structure (Rd). If the condition Sd ≤ Rd is fulfilled for each of the limit states, the structure is considered sufficiently safe (EC6, 2011).

This methodology, common in all types of concrete, metal and geotechnical structures, has not yet been reflected in historic masonry constructions, because these structures cannot be associated with a time period to ensure (with a limited probability of failure), the stability, bearing capacity and functionality of the structure; this term would be the total lifetime of the structure.

Thus, for the evaluation of the response of this structure, the known geometry and the most probable value of the properties of the materials (in the absence of more accurate data) have been considered. It is also known that the variable actions have little influence in many limit states, and for the permanent loads, their characteristic values are considered, since the structure clearly does not have compression problems, quite the opposite: in the case of vaults, increasing the axial is beneficial and the safety coefficient is not real (Ortega-Andrade, 1967).

Therefore, in the proposed methodology, actions will not be increased, nor will the materials be reduced as usual, but average strength values will be used and the structure will be checked under serviceability conditions, thus obtaining its intrinsic factor of safety.

### 3.3 Loadings

Before explaining how the various loads have been taken into account, it is worth noting that the predominant action in this type of structure is its own weight because the elements of the structure itself are massive.

The permanent gravitational actions are deduced from the geometry and the unit weights of the materials, which are obtained from the compiled bibliography (EC6, 2011). The numerical simulation was undertaken with the finite element software SAP 2000, suitable to encompass in an integrated manner the geometrical model, analysis and structural design (Quiroz-Torres, 2016). Because the geometry of the elements adapts to reality, the software employed already takes into account the self-weight of all the materials.

In the consideration of gravitational overloads, an attempt is made to answer the question raised about the quantification of the distributed or punctual overload that can be arranged on the roof in the current situation for a possible change of use and once the consolidation and waterproofing of the structure were carried out in 2012 (CYII, 2012).

**Figure 9:**
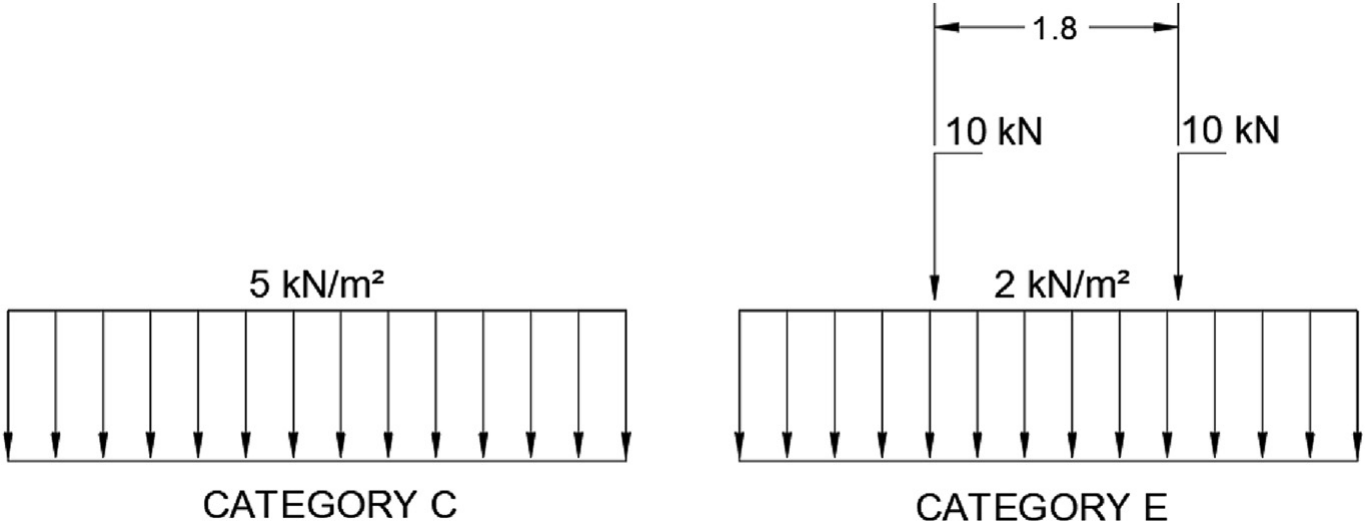
Loadings. Categories of use after (CTE, 2006) (dimensions in m).

According to the regulations about loads in building in Spain (CTE, 2006) for the use required for the roof, which is the opening as a public park, the category of use is C, public access areas, and within all subgroups, since the structure is subject to the most unfavourable load, which is 5 kN/m^2^ for distributed loads. The category E is also taken into account for light vehicle traffic and parking areas (total weight <30 kN), consisting of a distributed load of 2 kN/m^2^, in addition to a one-off load of 20 kN, broken down into 2 loads of 10 kN separated 1.80 m from each other. Therefore, both load cases (Figure 9) are studied non-simultaneously to obtain the response of the structure.

### 3.4 The strategy of analysis and methods

Depending on the complexity of the necessary models and the precision of the response (which is undoubtedly linked to the precision of the knowledge of the input data), the different “levels of analysis” are considered (EC6, 2011). At each level of analysis, a new set of equations to be met is introduced into the model.

At level 0, no condition is imposed on the structure and traditional dimensioning tools are included. Different construction procedures from the 16th century (Huerta, 2005) collect very similar rules in relatively distant geographical locations (such as Spain, Germany and Italy), which demonstrates that all these procedures have a coincident source in Europe from the previous centuries.

The equations that are imposed for the first level study are those of equilibrium. Starting from the geometry and known loads, and through simple and fast calculations, a first approximation for the solution is obtained. Logically, the hypotheses that are admitted in the calculation are very general and may not be entirely conservative.

At the second level, the analysis methods will impose two of the three groups of equations, in general, the equilibrium and the constitutive ones. It is a somewhat more rigorous problem with a greater need for data entry and also the calculations are complicated. One calculation is usually made at the structure level and another at the section level. The first can be merely elastic, to later perform a sectional calculation taking into account the non-linearity of the materials. One of the options is to carry out an analysis of the structure using equilibrium equations and a subsequent sectional analysis using interaction diagrams. As in the case of the first level, the solution is indeterminate.

For the third level of analysis, the amount of needed information is higher. At this level of analysis, the three groups of equations are imposed in the same calculation at the same time, namely equilibrium, constitutive and compatibility. This level is completely rigorous, although the calculation is also complicated, the solution obtained is unique. In this analysis, which is the one chosen for the structure under study, a single model is used. It is not possible to separate behaviour at the structural level on the one hand and sectional behaviour on the other. It works with tensions and not with efforts as in the previous cases. At this level, the most extended modelling techniques employ finite elements (chosen option), in which the generalized matrix formulation and damage theories can be highlighted. All of them are very powerful computing resources.

As a final for the justification of the adopted modelling approach, it should be noted that the main drawback of this type of calculation in our case is the double curvature in the geometry, which does not fit the use of bar (one-dimensional) or plate (two-dimensional) elements. Therefore, 3D simulations are needed.

### 3.5 Finite element method

A 3D finite element model is chosen to develop the present study, verifying the equilibrium equations, constitutive of the material and compatibility at the structural level. This is the best model that could be proposed without considering the non-linearity of the masonry neither in traction nor in compression. If the model does not generate tractions, or these are admissible in the fabric and are punctual, it can be considered valid. Otherwise, at least, it will be indicative of the failure modes.

The data needed to conduct the model are the geometry and the characteristics of the materials, previously presented and described in this paper (Table 1).

**Table 1:** Material properties following (Martinez, 2003).

Units (kg/m3, kN, m, kPa)	MASONRY	COBBLESTONE	GRANITE	FILL	CONCRETE	E. CLAY	GRADED	GRAVEL
Weight per Unit Volume	2000	2000	2800	2000	2400	331	1850	1800
Modulus of Elasticity, E	1,5e6	8e5	8e6	5000,00	3e6	1,6e6	1500,00	1000,00
Poisson, U	0,1	0,2	0,2	0,2	0,2	0,2	0,2	0,2
Shear Modulus, G	6,8e5	3,3e5	3,3e6	2083	1,3e6	6,8e5	625	417
Compressive Strength	30600	2000	1,4e5	48000	20000	2200	15000	10000

**Figure 10:**
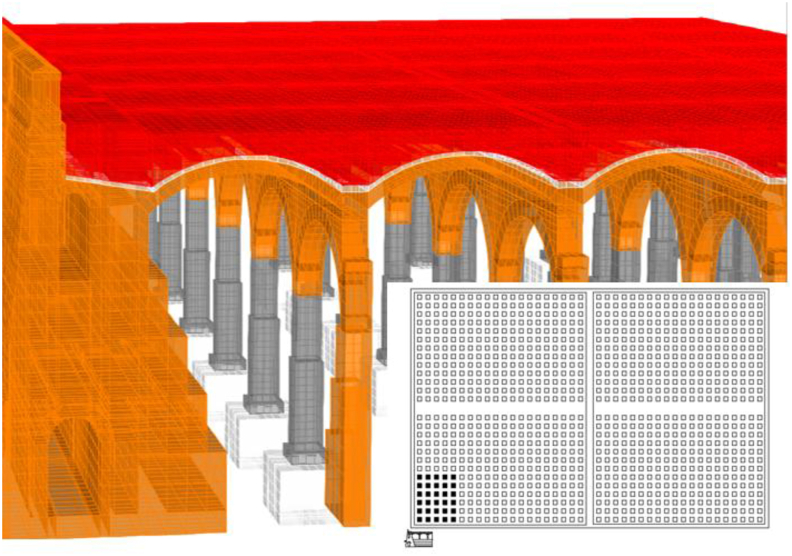
3D view of the model as original before actuation in 2012, including all simulated elements and materials: masonry in orange, cobblestone in white, fill in red and granite in grey. Sketch of the simuated fraction out of the total structure, in plan view.

**Figure 11:**
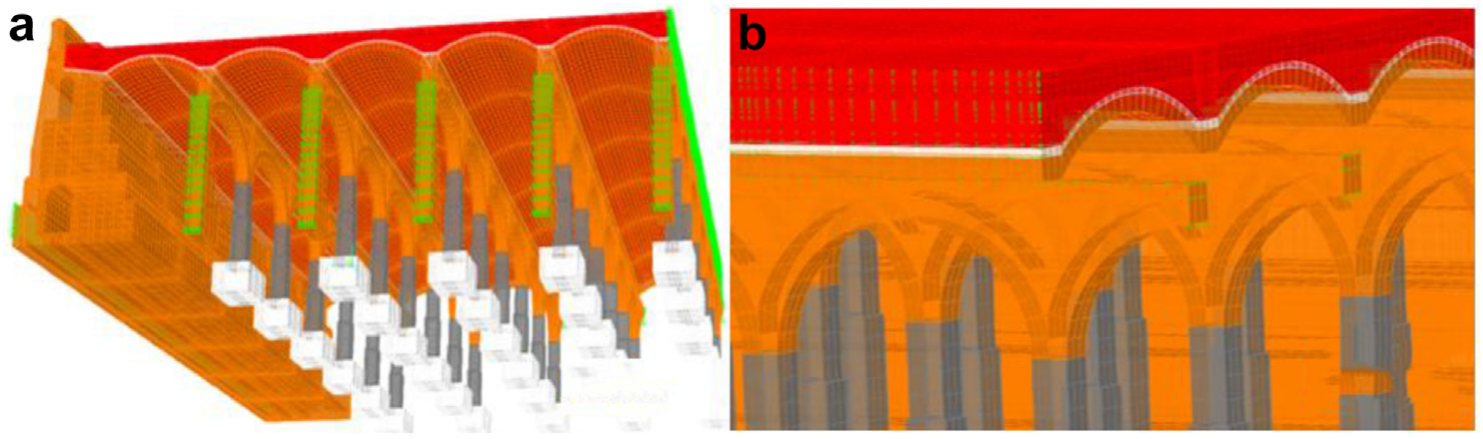
Boundary conditions in green. Longitudinal vault (a); Fills and lateral vault (b). Rest of colors meaning in Figure 10.

A linear (elastic) finite element model is proposed and, as long as it is verified that the tractions that appear are not significant, will help us to identify the working mode of the structure, as well as to quantify it so we can assess whether the failure of the materials is expected to happen or not. The modelling technique (Figure 10) consists of the use of solid finite elements, the main advantage of which is the direct determination of the shear stresses. In each node there are six degrees of freedom, corresponding to the three translations and three rotations of a three-dimensional problem that allows for simulating a continuous structure. Due to the excessive computational effort required for the complete study of the whole deposit, a representative part of it was simulated (Figure 10), which entails coercing the model at its ends in the direction of work of the vaults and arch. The fill has restricted displacements since it is confined by the structure (Figure 11(a, b)).

**Figure 12:**
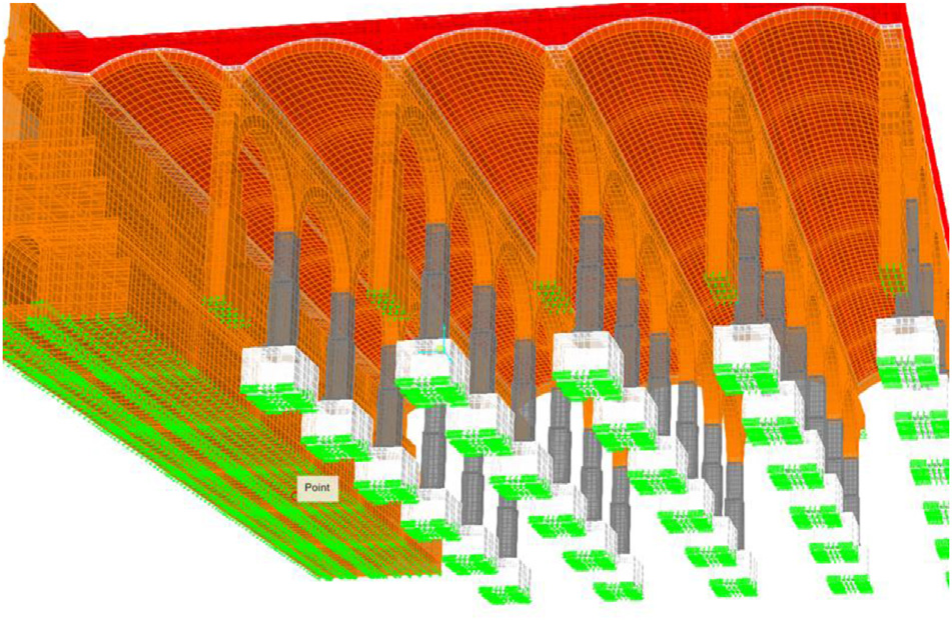
Boundary conditions under the footings in green. Rest of colors meaning in Figure 10.

The geotechnical characterization is deduced from the only geotechnical study available (CYII, 2011) and the vertical ballast module is adapted to the specific geometry of each foundation element (Figure 12).

### 3.6 Evaluation methodology for vaults and arches

#### 3.6.1 Equilibrium limit state

When the structure reaches the equilibrium limit state, that means that it stops fulfilling the equilibrium equations, forming a kinematic mechanism, which gives rise to a displacement of different rigid solids. Within this limit state, the following faults stand out: formation of a mechanism between arches or vaults and loss of balance of the counteracting system due to the displacement or overturning of any of the parts, in this case, mainly from the perimeter walls of the deposit.

#### 3.6.2 Instability limit state

In case the structure undergoes instability failure, the resistant properties (geometry and materials) of the faulty element and its deformability come into play. Two types of failure can occur, namely, in vertical elements, such as piers, and horizontal elements such as vaults or arches, the original shape substantially changes, especially in this case since the vaults are lowered.

The failure due to instability of vertical elements occurs when the eccentricities increase due to the effect of the deformations. It is not a worrying fact in the structure under study since in this case the cells are loaded symmetrically on both sides for the majority load. Special attention will be paid to the effect of an eccentric overload, although it is expected that its contribution might be negligible compared with its own weight, there will be no problems of this type.

This failure is more common in structures with lateral vaults, especially when there is a difference between the heights of two adjoining naves.

The instability of horizontal elements is not very common and is due to construction defects. After so many years of service, this risk can be ignored, but its analysis might be convenient.

In the first place, this failure can only occur in lowered vaults, since this type of vault transmits significant horizontal loads to the counterbalance system. When these efforts cause excessive deformation due to the rigidity of the lateral containment system, the failure of the vaults takes place by means of losing their shape.

#### 3.6.3 Failure due to the material reaching its limit strength

**Figure 13:**
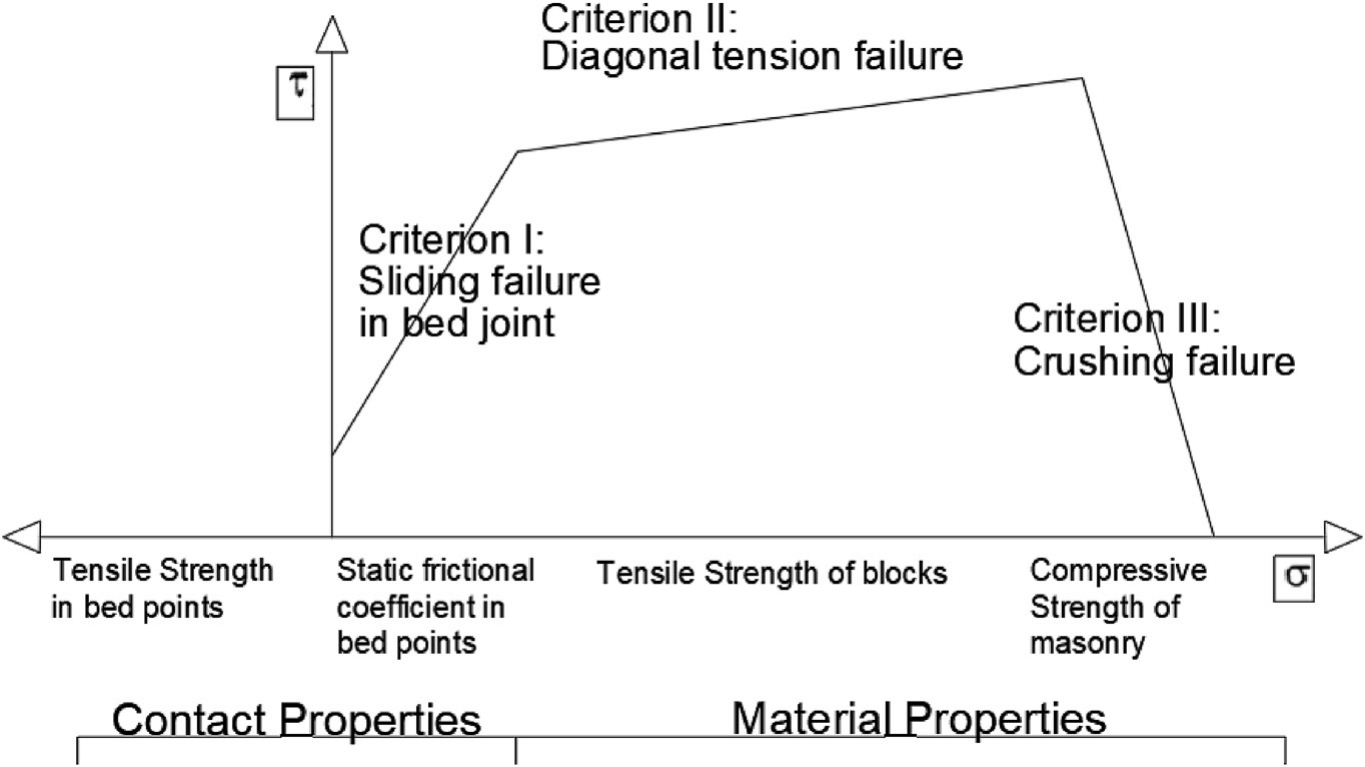
Mann and Muller depletion diagram. Failure patterns and cracking type that occurs according to the type of failure of the fabric after (Mann and Müller, 1982).

Within failures due to material depletion (Riddington and Ghazali, 1989), various failure modes can occur (Mann and Müller, 1982), as explained in Figure 13, according to the Mann and Müller depletion diagram.

The failure due to compression is represented in the third branch of the Mann and Müller diagram. This type of masonry failure is characterized by a pattern of cracking parallel to the compressive stress. In the second branch of the diagram, failure by compression and shear is represented, and it is characterised by a cracking pattern that develops through the pieces. Thread separation failure is due to compression and shear runout and is represented by the first branch of the diagram. This type of failure is characterised by a cracking layout that develops exclusively in the clotheslines. As the arches and vaults do not have superimposed threads, it is difficult to obtain this effect. In fact, the perpendicular configuration of the vault pieces with respect to the appearance of tangential stresses would compress the pieces and joints, precisely perpendicular to them if the perfect funicular had been achieved. Shear failure, like the previous one, is represented by the first branch of the Mann and Muller diagram and is characterized by slippage between pieces.

#### 3.6.4 Durability limit states

The durability limit state is validated if the time required for the aggressive agent (physical or chemical actions other than the loads used in structural analysis) to produce a significant degradation of the mechanical characteristics of any of the structural elements is greater than the design value of the useful life. In the end, the limit state of durability ends up reducing the strength of the material until one of the previously mentioned failure mode takes place.

There is no doubt that the consolidation and waterproofing action carried out in 2012 improved the conditions for the structure, avoiding attacks by water seepage, the appearance of new vegetation, etc.

**Figure 14:**
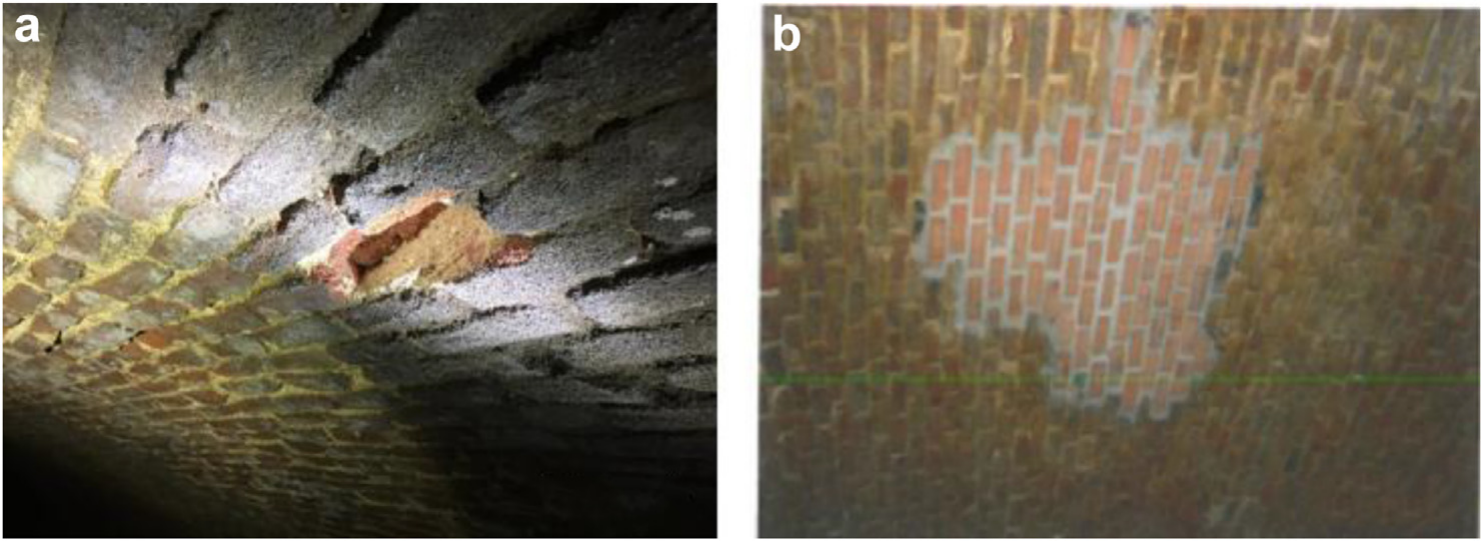
Detachment of materials from the vault (a) and repairs to vaults after the 2012 action (b).

In a previous inspection of the structure, it was concluded that its level of conservation is good (Intemac, 2011), and this is corroborated by the visit made as part of the present research. However, after a visual inspection of the deposit, some deteriorations such as those due to physical processes (humidity, salt attacks, loss of materials...) were identified (Figure 14(a, b)).

**Figure 15:**
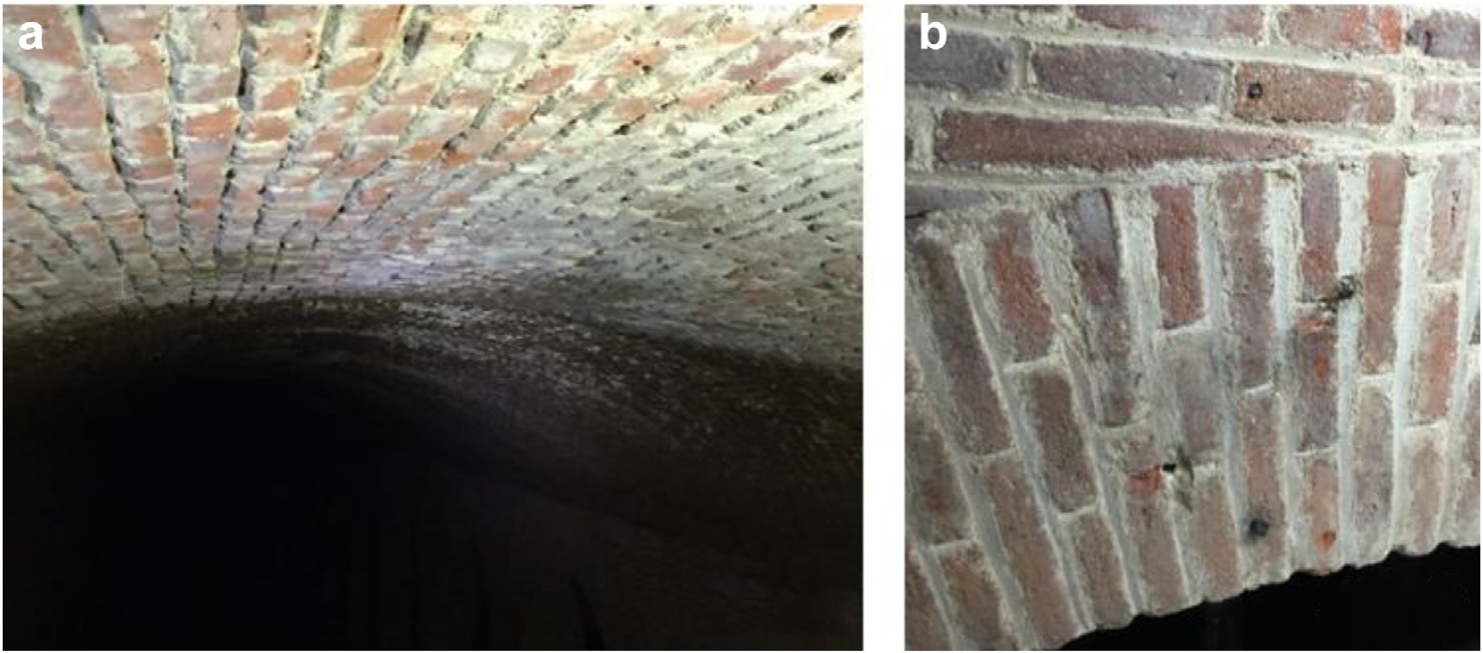
Lack of materials in the vault (a) and insignificant oxidation processes (b).

Alterations caused by water, loss of binding material from the vault and processes caused by the oxidation of metallic elements were also detected (Figure 15(b)), in addition to saline efflorescence due to loss of section after the 2012 action, all processes caused by external agents (human beings, vegetation...) or even detachment in the vault caused by a shell (Figure 15(a)) found in the excavation during the action (CYII, 2012).

## 4 Results

One of the objectives of this study and the choice of a 3D model with “solid” type elements (Isla, 2017) was to quantify the structural action of all types of materials, taking into account the scarcity of available data, highlighting the need to test some or all of them in the future in order to draw more reliable conclusions. Three scenarios have been modelled and analysed. First, according to the original situation of the tank with a greater thickness of fill before the 2012 actuation. Also, the current situation with a lightened section after the 2012 actuation, and the future situation with the new possible proposal for a change of use, with the same current structural section and subjecting the structure to new loads.

### 4.1 Analysis of the original deposit according to the structure model before the 2012 actuation

**Figure 16:**
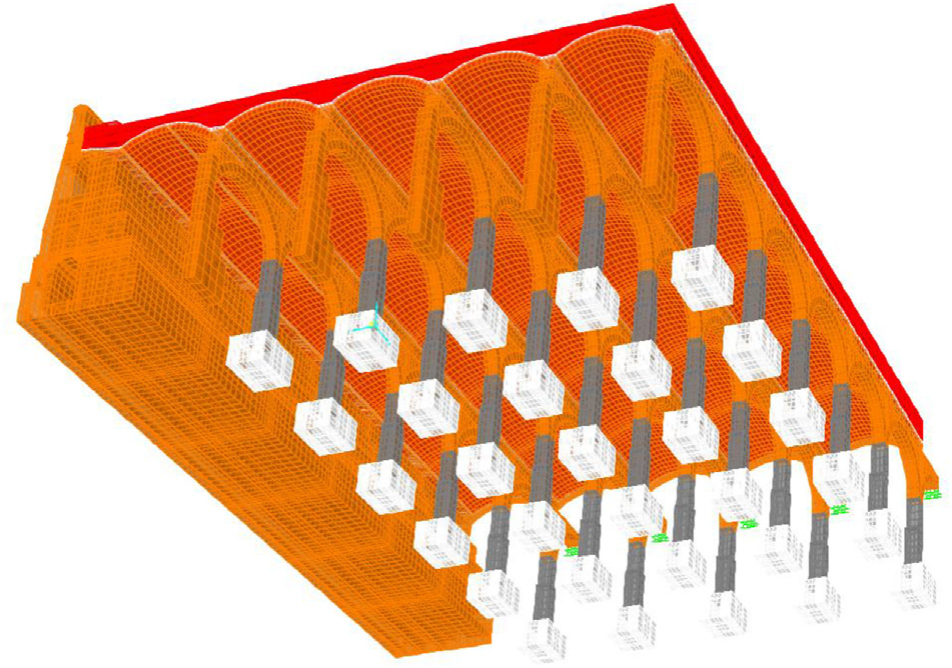
General view of the model developed (size 30 × 35 m2 of total size 208 × 138 m^2^) for the original situation where the red colour represents the landfill, the white the cobblestone used to cover the vault (10 cm) and also the foundation material, the orange colour is the masonry and the grey for the granite of the pillars.

**Figure 17:**
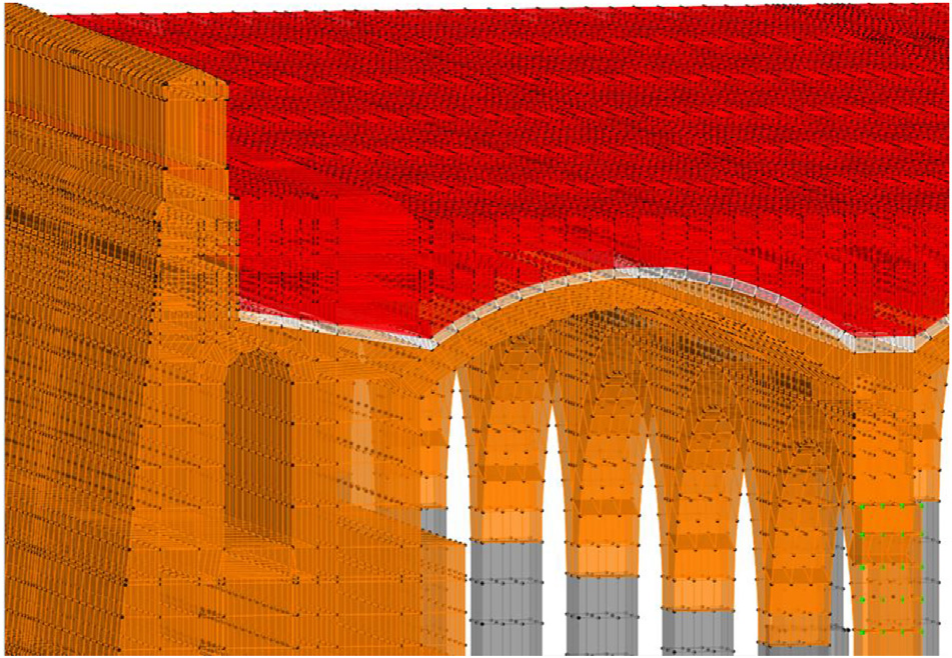
Model elements and nodes.

**Figure 18:**
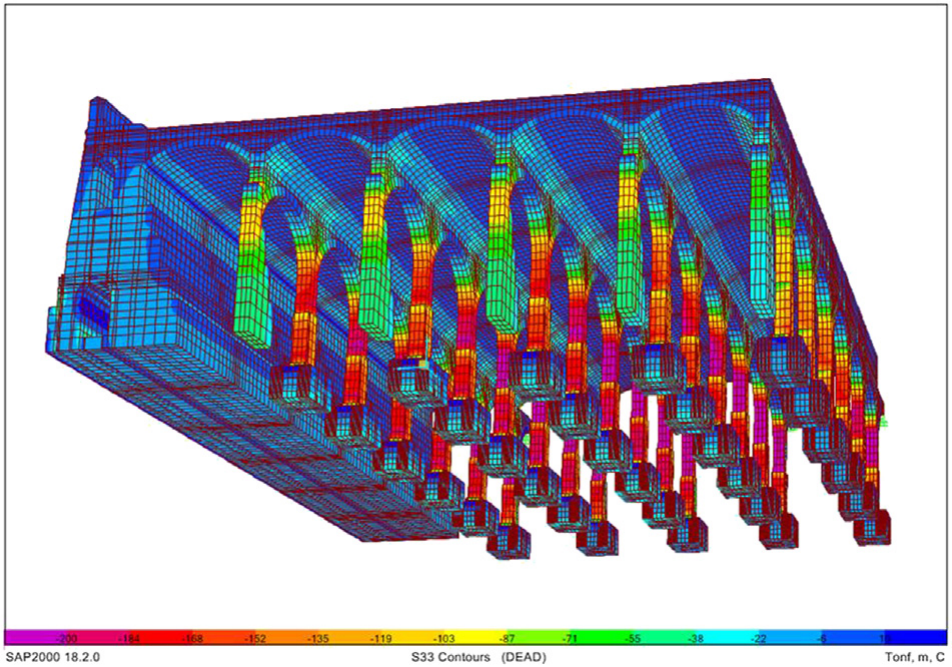
Normal Stresses in the original structure under gravity loads only. Units in Tons and negative values for compression.

**Figure 19:**
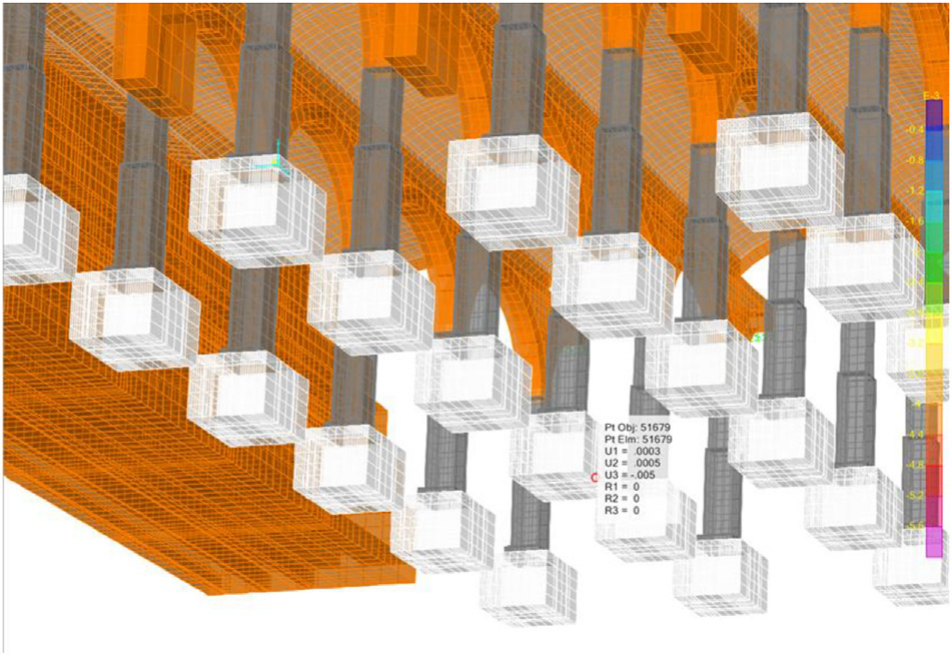
Foundation settlement.

A series of illustrations are attached as an overview of the model of the original structure, prior to the actuation which took place in 2012 (Figure 16) where the connection between elements simulates a continuous structure (Figure 17). Then, the results obtained are attached in a global form, such as the stress map of the whole tank (Figure 18). The foundation lowering is shown (Figure 19), considered equally in all the piles. The different structural functions of each element, infill, vault, arches, piers, and foundations, deserve a detailed and independent study. It is well known that they all contribute structurally and therefore each must comply with certain limit states. Such illustrations give a clear idea of the unambiguous analysis to be carried out, according to the data described above and bearing in mind that the uncertainty is large.

Only the permanent gravitational actions that are deduced from the geometry and unit weights of the materials (CTE, 2006) have been considered.

It is worth noting that only for admissible stresses on the ground, the weight of the water should be added to the full tank. This same action generates a hydrostatic thrust against the perimeter walls, but due to their rigidity (in some cases buried) they do not introduce relevant uncertainties at the superstructure level. Therefore, this load has not been considered, it does not affect the superstructure overloads that are really the objective of the study and the structure has been working correctly under this action since its construction.

The results obtained are studied globally, making clear the connectivity between elements, idealized as continuous (Figure 17).

Obviously, the different structural functions of each element, i.e. fills, vault, arches, piers and foundations, deserve a detailed and independent study. It is well known that they all contribute structurally, so each one must meet certain limit states. In addition, the scale order must be particularized for each element, since, although all are essential, each one works at a different tension level (Figure 18).

The admissible vertical stress of the fill and its distribution in the fill-coarse interface on the roof in which the own weight of the ground marks the trend are analyzed separately. Likewise, it is observed that for the shear stresses the order of magnitude is about 10 times less than the vertical normal stresses, which indicates that there will be no sliding problems on the contact surface between both surfaces. From the deformation obtained, it can be concluded that the fills are difficult to compress, although taking into account that the foundation settles 5 mm (Figure 19), the deformation of the fills will be between 1 and 2 mm depending on the thickness.

**Figure 20:**
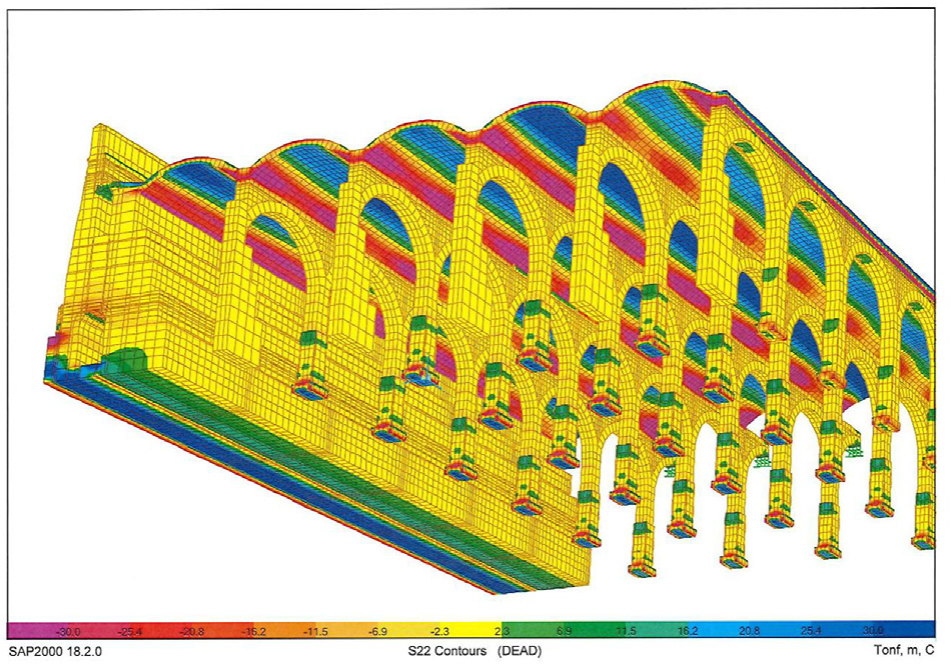
Tension map of the vaults of the deposit (Bottom view, Units in Tons and negative values for compression).

The results of the vault and masonry analysis indicate that the vertical stresses in the top and bottom fibre of the keystone masonry approach zero (Figure 20), reflecting the design optimization of the original structure. Compressions accumulate in the vault openings, clearly marking their operation. All the stresses in the masonry are compressive, clearly marking the tendency towards the upper fibre of the masonry-masonry composite of the pressure line of the structure.

The shear stresses in the masonry mark the performance of the structures in the horizontal plane, that is, clear compressions in the upper fibres and some tractions in the lower fibre. Clearly, the pure antifunicular is not reached, that is, the self-weight compression line does not pass exactly through the centre of masses of the cross section. Only about 10% of the vault section is under tension in the horizontal direction, but always below the permissible tensile stress according to the literature (Figure 14). In the horizontal direction, the masonry is compressed throughout its section at the key level. Due to the settlement of the foundation around 5 mm, we clearly see how the base of the vault lowers the same, that is, the frame and base are non-deformable, and the key shows a deformation of 1 mm, giving an idea of the rigidity of the whole.

The arches operate similarly as the vaults, reaching null tensions in the keystone and increasing the compressions towards their starts, where the maximum compression is concentrated in the masonry, before making the transition to the granite. Quantitatively, stresses for the outer fibres of the section, which are obviously more loaded than the central core at 1800–1900 kPa, have values lower than the recommended 2000–3000 kPa. As in the vaults, the tractions that appear in the lower fibre of the arches in the keystone are minor. Note that in the extreme arches the difference in differential settlements increases the tractions significantly, although this is not a real situation. It is very important to avoid differential settlements between foundations to guarantee structural integrity. Vertical deformations of around 0.5 mm are expected in the keystones of the arches, since the vertical displacements at the start of the pillars, and consequently at the foundations, is estimated as 5 mm.

The slenderness of the structure and its degree of hyperstaticity is also evident at the deformed level.

As for the results of the foundation analysis, the stresses in the foundation material (cobblestone) and consequently the compressions distributed per unit area that are transmitted to the ground are obtained in detail. Seen in detail, the transmitted tension is 260 kPa. It is worth noting that the rigidity of the foundation defined as cobblestone, makes the stress distribution not be homogeneous at the foundation level, being localised under the support. It can be inferred that in view of the data, new drilling may reflect the appearance of a rigid material (concrete) that will relieve the stress state. All the settlement observed in the pile, as we mentioned before, is due to the settlement of the foundations, considered equally in all the piles. In this way, we consider null the elastic deformation of the material that makes up the structure, for self-weight loads.

### 4.2 Analysis of the current tank according to the structure model after the 2012 actuation

**Figure 21:**
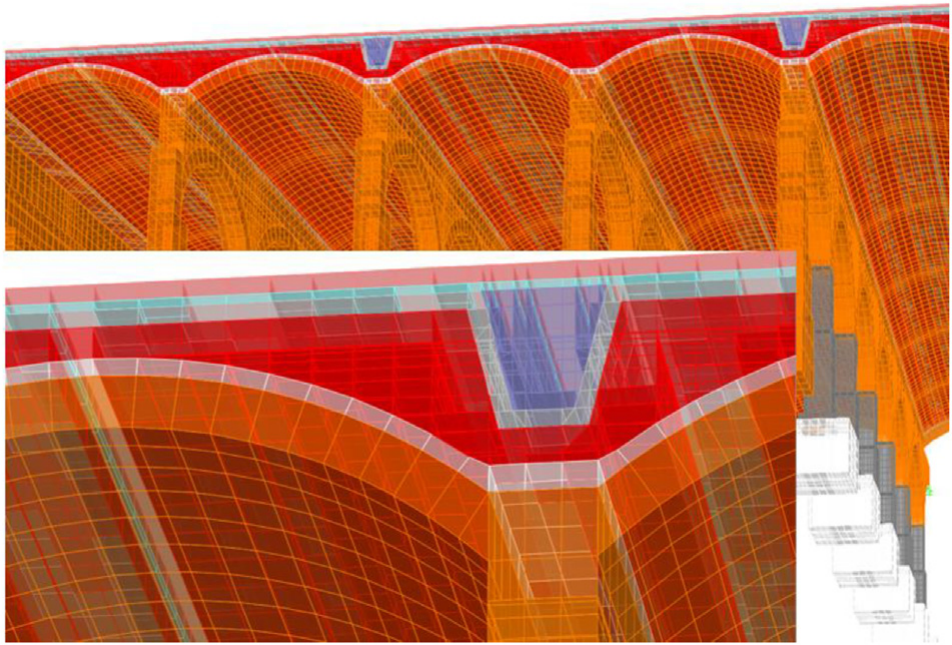
General view of the model and the connection between elements in current situation after 2012. Detail of modelling of masonry in orange, cobblestone in white, concrete in grey, gravel in blue, expanded clay in clear blue and graded in clear red.

As a peculiarity with respect to the original deposit, in the model of the current situation, it is worth highlighting the loss of fill (around 30 cm), in addition to its replacement by new materials (Figure 21), which shows a stress state of the fill similar to the original one, although the loss of material to allocate the drainage galleries, as well as the introduction of concrete compression and masses that gravitate on the souls of the channels, clearly compress the little fill under the drain, although not alarmingly. The concentration of vertical stresses on the constriction of the fill is evident, in some cases exceeding 40 kPa. The trend will be, once the structure is loaded, to compress under the ribs of the drainage channels more significantly than where the fill has not been disturbed. From the stress analysis of the concrete slab, it can be deduced that the concentration of compressive stresses in the nerves of the drain channels is considerable, which leads to concentrating the loads placed on it in those areas, modifying the distribution and the path of the stresses towards the columns.

As for the clay, load distribution is homogeneous, except in the drainage channels, where the sign of the stresses is inverted, that is, traction is achieved, although quantitatively insignificant. The tendency to hang the load from the webs of the drainage channel can be noted.

Although the structure was unloaded after the intervention (CYII, 2012), it is worth highlighting that the shear stresses clearly increase under the drainages, with which the trend to slip in the fill - cobblestone interface at that point increases. It is true that the concentration of vertical loads also increases in this area, but, in any case, special care should be taken with the geometric modification of the fills in this type of structure. The tension map that the filler throws has been clearly modified after the action.

The shear stresses are concentrated in the concrete slab on the fill, discharging it in terms of the aforementioned stresses. The clear distribution function performed by the concrete slab and subsequent discharge or focus of stresses in the drainage channel areas, obviously makes this slab work in practically pure tension, which makes us think that any addition of fibres would be very convenient. The problem is to quantify what level of stress appears horizontally, to specify that in the case under study it does not crack. It is well known that the addition of fibres modifies the non-linear behaviour of concrete, preventing or delaying the appearance of cracking in the concrete. Future testing could determine whether the concrete will remain without cracking after loading the structure or not. In this case, the deformation of the fills is nearly zero (0.1 mm), since the action of load distribution by the concrete slab causes the fills to remain elastically unchanged.

For the rest of the elements, no significant differences are observed with respect to the original situation.

### 4.3 Analysis of the current deposit to support new actions and land uses

The model used for the application of the loads is the same as that described in the previous point. According to the specified overloads (CTE, 2006), they are added non-simultaneously to the model, in addition to doing it in a semi-vault and semi-arch:

Type E overload on the structure (2 kN/m^2^+ 10 kN punctual) and Type C overload on the structure (5 kN/m^2^+ 7 kN punctual). After arranging the 2 kN/m^2^ in addition to the punctual load of the light truck, the structure is loaded with different provisions of type C overload, looking for all the possible lines of influence of the structure.

Once all the possible overloads defined have been defined, they are combined with the self-weight of the structure, in order to quantify the overall stress state, although it is already guessed that the self-weight will be the main applicant at roof level, although frames and supports will be more sensitive to the increase in vertical load.

The results indicate that the overloads applied to the model compress the vaults without introducing significant tractions in the key to the same, with which it seems that these elements are able to receive the normative loads, even with load alternation. Likewise, with regard to the shear stresses, the reached values take into account that the overloads are not very different from those of their own weight in the original situation.

**Figure 22:**
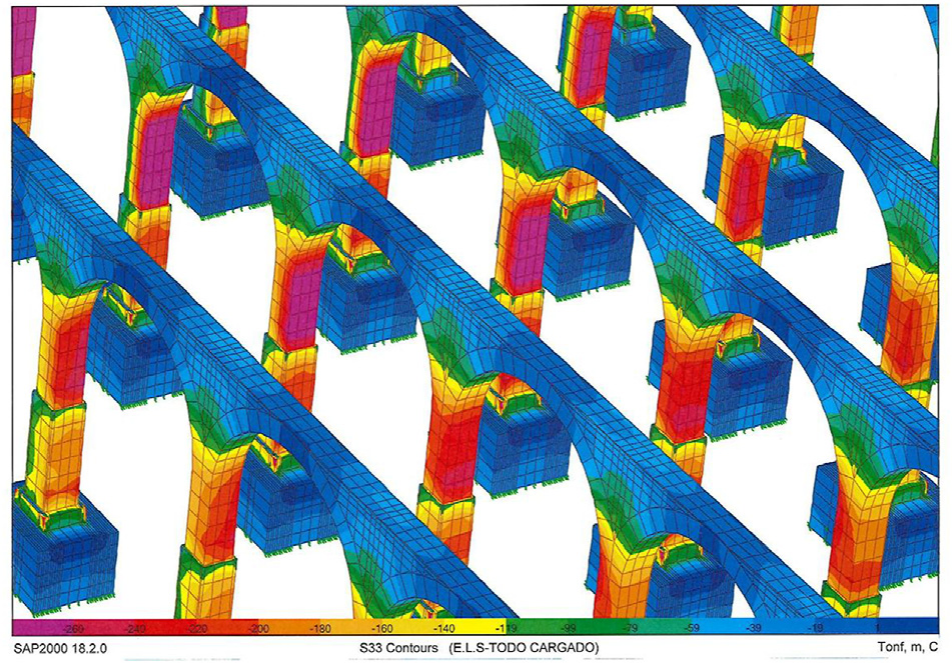
Normal Stresses in the arches, their starts, and pillars in the current situation with the new loads that are intended to be placed.

In the starts of the arches, the maximum compression is concentrated in the masonry (Figure 22), before making the transition to the granite. Quantitatively, it is valued for the outer fibres of the section, which are obviously more loaded than the central core at 2400 kPa.

The stress state of the foundations is around 280 kPa, slightly higher than the original ones, as we saw in the study of the original model. The foundation after the solicitation of permanent loads and overloads settles in the order of 5 mm.

## 5 Discussion

This work reveals the need to be very cautious regarding the stresses that are required in the future for historic structures such as the one described, and, as a basis for structural safety, not to exceed the original stress state. It should be noted that in terms of current resistance, the useful life of the studied deposit is exceeded, although they would not be applicable in this type of historical structures, since obviously many are still in use and with structural functionality today.

At the roof level, the structure was unloaded, after the waterproofing action after lowering the thickness of the ground by 30 cm and restoring it through various layers of concrete, clay and gravel. As can be deduced from the obtained results, the tension level of the vaults deviates from the pure antifunicular, appearing nonetheless null or with negligible tensile tractions in their key. Once the applied overload sweep was been performed, the resulting stress configuration was difficult to distinguish from the original one. As for the arches that support the vaults, the operation is similar to that of the vaults, that is, the structure is perfectly optimized in terms of the use of the material, since zero normal stresses are also reached in the keystones of the arches and punctual tractions are not very significant when we talk about the tangential ones.

From the study of the loads to be placed over the structure for its new uses, once it is loaded and for the overload hypothesis of 5 kN/m^2^ Kg/m2, compression values for the same section of 2400 kPa are reached, with which the limit of the recommended capacity is reached, surpassing the normal stress values of the primitive structure and also taking into account that the hypothetical safety coefficients that were established in the past, understood as we understand them nowadays, have been decreasing due to the age of the structure.

As for the foundation, in the case of new studies, it is recommended to check the material they are made of since nowadays there seems to be certain discrepancy between whether it is masonry or concrete; in addition, it is necessary to know or better characterize the terrain on which the structure sits since any settlement would be very detrimental for the structure. For this reason, we believe that it is necessary to prudently avoid transmitting more loads to the ground than it supported in the past, since it is assumed that the settlement in 150 years is already consolidated.

The apparent good condition of the structure should be highlighted. However, it does not seem appropriate to contemplate changes in uses that differ from the current one, since it would entail an increase in loads that is not compatible with the current proposals to create an urban park on its roof.

The foregoing is a critical point as the tank is in use and supplies a large part of the city of Madrid, hence maintenance and cleaning work on the structure must be carried out to advance in its detailed review and diagnose it conveniently. At the structural level, it is essential to know the most significant parameters of the materials, as well as the supporting ground of the structure that allows us to approach the problem and validate the characteristics used according to the specialized bibliography with which this study has been carried out. It is proposed to carry out representative surveys obtaining samples of the foundation and foundation ground; as well as non-destructive tests by means of ultrasound of the granite that makes up the stacks of the deposit.

From this analysis, it was concluded that the structure could withstand the loads currently set by the regulations in Spain for public spaces, that is, 5 kN/m^2^. While waiting to carry out a complete study of pathologies, tests, characterization of materials and the ground, it is recommended not to load the current structure with a distributed load greater than 3 kN/m^2^ so as not to exceed the stress state that it has supported up to now.

The constructions can have other uses: a new bridge in Alcántara to liberate the Roman bridge from traffic (Instituto de Ingeniería de España, 2021) can be an example of how the technique is beginning to be considered a heritage asset (Roca Frabegat, 2011). Also, It is necessary to promote the coexistence of heritage with growth or rather with the redevelopment of cities for the pursued environmental and social sustainability (José María García Mezquita, 2007).

It is worth pointing out the need for greater awareness of heritage in public administration. Certain sectors of civil engineering and the heritage field are promoting interest in the approval of a national civil engineering heritage plan, coordinated by the State administration and with the active participation of the regional administrations (Foro, 2019). This plan would highlight the importance of this type of cultural legacy, which is particularly rich in Spain, and the different ways of managing it (Ministerio de Cultura y Deportes, 2021). Having external advisors, both national and international could be an effective framework for reflection on objectives, methodologies, and procedures. This is one possible avenue but not the only one. The orchestration of a civil society that is more informed and more sensitive to the management of heritage elements included in sustainable development is the main challenge and the best guarantee that the actions correspond to society's aspirations.

## 6 Conclusions

Due to the nature of the structure of historical constructions such as the 19th century underground tanks in Madrid, and in particular, the so-called Major Deposit or Second Deposit of the Canal de Isabel II, in every action on these old infrastructures it seems convenient as a good starting point, not to exceed the tensional state with which these structures have been working for centuries, in order to guarantee their conservation.

A theoretical model of these problems has to be made according to the data provided and considering that there are uncertainties that significantly influence the achieved solution.

An exhaustive work of compiling the available information and its careful study is decisive in historical works, in order to know in as much detail as possible, the history of loads, materials, and actions on it. This entails an additional difficulty, such as getting access to tanks such as the one studied in this paper, which is closed to the public because it is in use and requires permission from the owning administration. The first author of this work visited the tank to undertake a careful inspection and concluded that it was in good condition, although, without a doubt, the structure is sanctioned by its use and solicitations during its 150-year history.

This research presents the case study that can be of application to similar historical infrastructures, in order to assess and evaluate whether new uses and actions can be conducted in them while they are preserved and their integrity is guaranteed. Three scenarios have been analyzed according to the original situation of the deposit with a thicker fill, the current situation with a lightened section after the intervention and waterproofing in 2012, and the future to accommodate new uses, with the same structural section, subjecting the structure to certain overloads.

As mentioned during the drafting of this document, and due to the importance of the implementation of new uses, is proposed as future research work a specific and detailed study of pathologies must be carried of this type of infrastructure, especially hydraulic works. At present, research and diffusion work is infrequent, and in the case of the tank, in particular, it should be carried out in the future before any decision is taken on the use to be made of it. A major limitation of these infrastructures is that they are in use, full of water, and supply large areas of big cities. It is also their accessibility for inspection work and for carrying out the material and ground tests that must be performed to delimit the uncertainties inherent to this type of structure. This has been one of the major limitations of this study, as well as the size of the infrastructure, which makes it practically unfeasible to characterize the materials with complete certainty, making it impossible to know the real stress state of the structure.

This study shows an example of the conservation of the historical legacy in front of economic and political perspectives that push for other solutions that are not always appropriate from the point of view of preserving the heritage.

## Declarations

### Author contribution statement

María Jesús Rosado-García: Conceived and designed the experiments; Performed the experiments; Analyzed and interpreted the data; Contributed reagents, materials, analysis tools or data; Wrote the paper.

Susana López-Querol: Analyzed and interpreted the data; Contributed reagents, materials, analysis tools or data; Wrote the paper.

Daniel Crespo Delgado: Contributed reagents, materials, analysis tools or data.

### Funding statement

This research did not receive any specific grant from funding agencies in the public, commercial, or not-for-profit sectors.

### Data availability statement

Data included in article/supp. material/referenced in article.

### Declaration of interest's statement

The authors declare the following conflict of interests: Susana López-Querol [an associate editor for Heliyon].

### Additional information

No additional information is available for this paper.
